# Diabetes Self-Care among South Asians in Western Countries: A Systematic Review

**DOI:** 10.1007/s10903-025-01825-4

**Published:** 2025-11-20

**Authors:** Saranya Puzhakkal, Barbara Conway, Syed Tabish Razi Zaidi, Chia Siang Kow, Syed Shahzad Hasan

**Affiliations:** 1 Department of Pharmacy, School of Applied Sciences, University of Huddersfield, Huddersfield, UK; 2 School of Pharmacy and Pharmaceutical Sciences, University of Tasmania, Hobart, Australia; 3 School of Pharmacy, Faculty of Medical and Life Sciences, Sunway University, Kuala Lumpur, Malaysia

**Keywords:** Immigrants, Diabetes, Self-care, Self-management, South asian, Type 2 diabetes, Western countries

## Abstract

Diabetes self-care is a patient-driven approach to managing diabetes, supported by healthcare professionals, that aims to empower patients to reduce long-term complications associated with the disease. This is particularly important for South Asians, who are among the populations most affected by diabetes. This mixed-methods systematic review synthesises both qualitative and quantitative evidence on diabetes self-care among South Asians living in Western countries and presents an integrated framework covering key areas for optimal self-care. The review followed PRISMA guidelines, and the protocol was registered with PROSPERO. Seven databases were searched for both qualitative and quantitative studies published between January 2000 and December 2024 to provide a comprehensive understanding of the evidence. The Joanna Briggs Institute critical appraisal checklist for quality assessment and the Risk of Bias V2 tool for trials were utilised, with data integrated through the Pillar Integration Process (PIP). Twenty studies met the inclusion criteria and were incorporated into this review. Most qualitative, cohort, and cross-sectional studies met over 70% of quality criteria, and all four randomised controlled trials demonstrated a low risk of bias. Qualitative findings highlighted the significance of culturally relevant education and support, while quantitative studies showed improvements in diabetes outcomes through educational and physical activity interventions. Common barriers included difficulties with self-monitoring, adherence to dietary guidelines, and implementing lifestyle changes. The PIP analysis identified three core pillars: providing adequate diabetes education, involving patients in care planning, and encouraging daily physical activity. Culturally tailored education, improved healthcare access, and patient-centred interventions are vital for enhancing self-care among South Asians. As this population’s presence in Western countries increases, broader participation in self-care programmes can promote better integration and health outcomes.

**Supplementary Information:**

The online version contains supplementary material available at https://doi.org/10.1007/s10903-025-01825-4.

## Introduction

Approximately 537 million adults aged 20 to 79, or one in ten, have diabetes. This number is projected to rise to 643 million by 2030 and 783 million by 2045 [1]. The increasing prevalence of type 2 diabetes mellitus (T2DM), its impact on morbidity, quality of life, and healthcare utilisation, makes ita global public health concern [2, 3].

People of South Asian heritage include those of Indian, Pakistani, Sri Lankan, or Bangladeshi descent; studies have shown that these groups have a higher risk of Type 2 diabetes [1]. Individuals from neighbouring countries, such as Nepal, are also considered South Asians [4]. Nearly 2 billion people worldwide are South Asians, and despite having a low average Body Mass Index, the prevalence of T2DM remains significantly higher among them compared to other ethnic groups [5]. One plausible reason for South Asians’ increased risk of diabetes is their tendency towards insulin resistance, which is exacerbated by high levels of visceral adiposity [5–7]. The diets of South Asians are mainly rich in high-glycemic foods, including polished white rice, refined-flour flatbreads, potatoes, and deep-fried snacks, all of which contribute to an elevated glycaemic load [8].

Research in diaspora nations, where people of South Asian origin have lived for generations (such as the United Kingdom and South Africa), has found that the prevalence of type 2 diabetes mellitus (T2DM) remains higher than that of the native population [9–13]. Compared to other ethnic groups, South Asians living in England are disproportionately affected by diabetes, with nearly half a million diagnosed and a quarter of a million with prediabetes [14, 15]. High insulin resistance, impaired insulin secretion, and increased ectopic fat deposition are postulated as key contributors to the development of diabetes in South Asians. Unique influences from social networks, religious beliefs, and limited physical activity also contribute to the increasing risk of diabetes [15].

As diabetes is a lifestyle disorder, self-care is one of the most crucial components in managing the condition [16]. Additionally, cultural, religious, and social practices often hinder effective diabetes management [17, 18]. Moreover, living overseas presents several challenges, including language barriers, limited access to healthcare services, and poor baseline health literacy [19]. This review aimed to analyse the self-care behaviours and practices of people of South Asian origin in Western countries and to identify effective self-care strategies in diabetes management.

## Methods

This systematic review followed the Preferred Reporting Items for Systematic Reviews and Meta-Analyses (PRISMA) guidelines (2020) [20] and was registered with PROSPERO (Reference No. CRD42024556002).

### Scope of the Review: Eligibility Criteria

The primary investigators (SP and SSH) screened titles and abstracts of articles reporting on self-care skills and practices among South Asian immigrants living in developed Western countries, published between January 1 st 2000 and July 2024. Original research articles with experimental and observational designs, such as cross-sectional, cohort, case-control, and randomisedcontrol studies, were included. Systematic reviews, meta-analyses, conference presentations, andletters ofcorrespondence were all excluded from the review. Only articles published in the English language were included.

The Sample, Phenomenon of Interest, Design, Evaluation, Research type (SPIDER) framework, designed for qualitative and mixed-methods systematic reviews [21], was used to structure the search strategy and inclusion criteria, as shown in Table S1 [6]. It is similar to the PICO framework used for quantitative studies.

### Information Sources

Medline, EMBASE, Cochrane Controlled Register of Trials (CENTRAL), PsycINFO, Cumulative Index of Nursing and Allied Health Literature (CINAHL), Clinical Trials (clinicaltrials.gov), and International Clinical Trials Registry Platform (ICTRP) were searched from January 1, 2000, to July 2024. Various text words and indexed terms related to self-management, self-care, and type 2 diabetes mellitus were searched. References of included studies were manually screened to identify additional articles. The supplementary file contains the comprehensive search techniques and alternative search terms for each database.

### Searching

The keywords used in different combinations for the search were ‘Type 2 diabetes or type 2 diabetes mellitus or T2DM or NIDDM or non-insulin dependent diabetes mellitus’, ‘self-management or self-care or self-regulation or self-monitoring’, ‘South Asian or Asian or Bangladeshi or Bengali or Gujarati or Indian or Srilankan or Punjabi’, ‘British South Asian’ and ‘South Asian immigrants. After screening the titles and abstracts, we excluded studies irrelevant to the review’s aim. The full texts of the remaining studies were reviewed to determine their eligibility.

### Study Selection

Studies of human participants with type 2 diabetes, regardless of age, were included. The assessment included self-care practices such as a diabetic diet, self-glucose monitoring, medication adherence, exercise, etc. RCTs were included to examine the effectiveness of self-care improvement interventions, including education, lifestyle modifications, and participant-directed interventions tailored to individual needs. Qualitative studies exploring similar concepts of diabetes self-care were included. In addition, qualitative studies that analysed the role of culture on self-care were also considered. We carefully reviewed all studies, particularly those using mixed-methods designs, to determine whether separate analyses were provided for South Asians. When such analyses were available, we included them in our review.

### Data Extraction Process

After sorting the studies according to the selected criteria, the data were input into a Microsoft Excel spreadsheet. The data extracted from the studies were the following: sample size, population, place of study, study design, study duration, how long being an immigrant, age group, gender, number of years being diagnosed with Diabetes Mellitus, whether translation was provided during the interview, concomitant diseases, self-care description, area of focus and significant findings.

Studies were separated based on their methodology, whether qualitative, quantitative, or mixed-methods and separate data extraction tables were created.

### Western Countries Definition

The term’ Western countries’ was used consciously, despite other widely recognised terms such as ‘high-income’ or ‘developed’, because of its relevance to migration and settlement contexts. These countries generally provide legal and social pathways for permanent settlement [22, 23]. Beyond income classification [24], the term reflects liberal values of human rights, equality, and integration [25, 26]. These sociopolitical factors influence migration decisions, with Western nations serving as distinct destinations for long-term settlement [27].

### Risk of Bias and Quality Assessment

The Joanna Briggs Institute (JBI) critical appraisal tools were used for qualitative, cross-sectional, and cohort studies, which contained 10, 8, and 11 items, respectively [28]. Each item has four options: ‘Yes’, ‘No’, ‘Unclear’, and ‘Not applicable’. Each paper included in this systematic review was analysed using this tool to determine how many items were fulfilled. Based on the results, stacked bar charts were developed for each study type.

The Cochrane Risk of Bias V2 (RoB V2) assessment scale was used for randomised controlled trials [29]. The RoB V2 framework utilises a predefined set of bias domains to focus on various aspects of trial design, conduct, and reporting. This tool includes items on the randomisation process, deviation from intended interventions, missing outcome data, measurement of the outcome, selection of the reported results, and overall bias.

Each stage of the systematic search and quality assessment was initially conducted by the first reviewer (SP) and subsequently validated by the second reviewer (SSH). Any discrepancies were resolved through a consensus process.

### Mixed-Methods Data Integration

The Pillar Integration Process (PIP) integrates common concepts identified in qualitative and quantitative data. This step is generally omitted in many mixed-methods systematic reviews and original studies. PIP is a data integration method that answers questions by combining qualitative and quantitative data from multiple studies to systematically identify key themes (pillars) across datasets [30]. By merging two distinct datasets, PIP ensures a structured approach to integration, providing comprehensive answers to research questions [30].

## Results

### Search Results

**Fig. 1:**
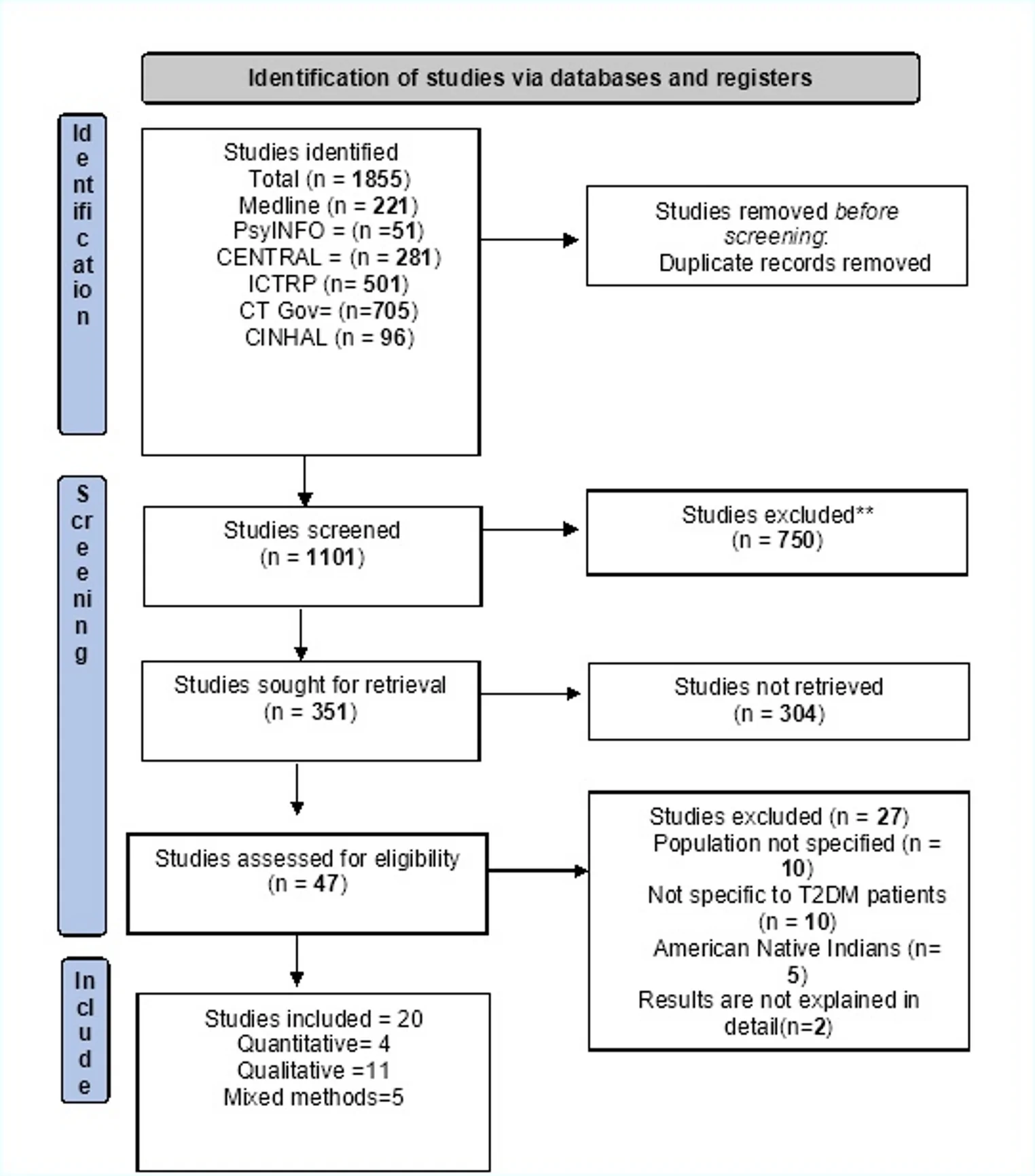
Study selection process – PRISMA

Out of the initially shortlisted 765 articles, 345 were excluded as duplicates. Another 246 were excluded after screening the titles, and a further 127 were excluded after reading the abstracts. Twenty-seven more articles were excluded after reading the full text for various reasons (see Fig. 1). Five studies conducted among American native Indians were also excluded. Twenty studies met the inclusion criteria and were included in this review.

### Description of Included Studies

Among the 20 included studies, four were quantitative [31–34], comprising three randomised controlled trials and one cohort study. Five studies employed mixed-methods approaches, providing both quantitative (observational) and qualitative data [35–39]. Eleven studies were qualitative [18, 19, 40–48] (see Tables 1, 2 and 3). Zhang et al. [48] conducted a relevant study on five ethnic groups living in New Zealand; however, only data about the Indian population were extracted and included in this systematic review. Of the four quantitative studies, two were conducted in the UK [31, 32], one in Canada [33], and one in the US [34]. The majority of qualitative studies were conducted in the UK (8 of 11). Among the five mixed-methods studies, two were conducted in the UK [38, 39], and one each in Australia [36], Italy [37], and the US [35]. These five mixed-methods studies primarily provided qualitative data.

**Table 1:** Summary of interventional studies

Study, Year, Country	Sample, Population, Translation provided or not	Study Design & duration	Age, Male/Female	Number of years since diagnosis	Self-care/management focus	Self-care/management description	Summary of main findings
Natesan A, Nimbal VC, Ivey SL, et al., 2015, US [28]	28 participants: Indian, Pakistani, Nepali, Bangladeshi, Sri Lankan	RCT 8-week pilot study	18–85, F-28	NM	Physical activity (Exercise)	Two one-hour Bollywood dancing lessons were provided twice weekly during the intervention. The primary outcome was HbA1c change. The effect of attendance on this outcome was also investigated.	HbA1c decreased in the intervention group from baseline, but it increased non-significantly in the usual care group. In comparison to those who attended fewer sessions (+ 0.86 kg (0.71 kg)), those who attended at least 10 of the 16 sessions saw a statistically significant weight reduction (− 0.69 kg (0.76 kg)). Compared to standard care, these findings suggest that culturally appropriate dance is an effective exercise intervention for HbA1c control.
A. Vyas et al., 2003, UK [25]	211 participants: Pakistan, India, Bangladesh or Sri Lanka; translation provided	Pilot parallel-group trial, 1 year	55.4, M-114, F-97	47.1	Education	Working with general practice staff, a diabetes specialist nurse, dietitian or chiropodist invited patients to four or more rotating visits per year. Patients’ and practice scores, awareness, and self-management of diabetes were assessed through an interview utilising a questionnaire at baseline and the 1-year follow-up. The responses were turned into educational resources that were utilised in the intervention.	The knowledge about diabetes and self-management was not adequately transferred by secondary or primary care support. For South Asian individuals with diabetes, primary care clinics run by specialists had no impact on knowledge or awareness or self-management between baseline and one year. Different clinician/patient information exchange means were developed for diabetes in this South Asian group.
K. Hawthorne, 2001, UK [26]	105 Pakistani women; translation provided	RCT, 6 months	52–57, F-105	L:7, IL:6	Culturally appropriate education	A link worker provided structured one-on-one diabetes health education using picture flashcards as visual assistance.	Nearly everyone improved their knowledge, and glycaemic control improved. Peers who were not formally educated did not show improvement in either area.

**Table 2:** Summary of observational studies

Study, Year, Country	Sample size, Population, Translation Y/*N*	Study Design, Duration	Age group & Male/ Female	years with DM & immigration years	Self-care/management focus	Self-care/management description	Summary of main findings
T.S.Tang et al., 2022, Canada [27]	114 India, Diaspora, & other South Asian; translation provided	Single cohort pre/post, 2 Inter, 1 &3 months	64.5, M-54, F-60	14.39	Participant-directed education	Twelve weekly sessions of short-term, group-based diabetes self-management education and support (DSME/DSMS) were part of the three-month intervention. The program consisted of six biweekly diabetes education meetings, led by a diabetes educator and a peer leader, and six biweekly diabetes support sessions, also led by a peer leader. Each DSME lasted 2 h (12 total) and was led by a diabetes educator and a trained peer leader who guided questions and facilitated discussions.	Participation in a peer-led and professional intervention designed for South Asian adults with type 2 diabetes is linked to better health outcomes, including improved glycaemic control.
Wan et al., 2022, Australia [30]	18, Indian, Yes	Mixed-method study, NM	40–63, M-13, F-5	8, 30 yrs	Dietary management	The average of three days was used to assess nutrient intakes and the quantity of servings in each food group. According to national standards, the recommended serving sizes of the five adult food groups were utilised as reference values for managing diabetes.	The median macronutrient compositions were 14.1% protein (IQR: 11.9–15.9%), 35.9% fat (IQR: 31.4–42.2%), and 48.6% carbohydrates (IQR: 41.9–51.9%). The median number of servings consumed by the five food groups did not meet the national dietary requirements. In terms of food intake containing carbohydrates, the majority of individuals consumed less grain (77.8%), fruit (88.9%), and dairy (94.4%) than the recommended daily intake of six servings of grain, two servings of fruit, and 2.5 servings of dairy for adults.
Piombo et al., 2020, Italy [31]	55, Bangladesh, Morocco, Algeria, Tunisia, Egypt, Yes	Mixed-method study, 2 Follow-ups, 3 &6 months	22–65, M-49, F-6	NM, 11.4 yrs	Culturally tailored dietary intervention	The dietitian created a personalised food plan tailored to the patient’s cultural background. The diabetologist chose patients based on their baseline age and country of origin. The nutritionist recruited and interviewed individuals with diabetes. The data gathered from the baseline questionnaire (pre-questionnaire) enabled the tailoring of each nutritional profile to the patient’s lifestyle and socioeconomic determinants of health.	Cereal, meat, and potato intake improved significantly at follow-up, and each patient had a significantly higher number of appropriate eating habits. Support from transcultural mediators received 90% good ratings. A transcultural intervention based on clinical and socio-cultural criteria, which takes into account patients’ lifestyles, has a positive impact on adherence to dietary control.
Patel et al., 2016, London, UK [33]	10, Indian, No	Qualitative study, Thematic analysis, 1	Mean 61, M-8, F-2	NM	Knowledge about good and bad foods for diabetes	For the pile sorting exercise, participants received 10 cards, each with a distinct food item written on it. They were asked to classify the ten items into two piles: those they believed were beneficial for people with diabetes and those they considered unhealthy.	Participants in the pile-sorting task demonstrated their understanding of which foods are suitable for individuals with diabetes and which are not. However, some individuals didn’t seem motivated to stop eating soft beverages, ghee, and white sugar. Among the factors contributing to this were their traditional Indian cuisine and culture. Having grown up on this diet, many people found it challenging to break away from it.
Patel et al., 2015, North West England, UK [32]	67, Indian, Pakistani, Bangladeshi and Nepali, No	Mixed-method study, 1 interview	Mean 61, M-36, F-31	0–10 + yrs, NM	Illness beliefs, fatalistic beliefs, Diabetes self-care, Physical health	The 9-item Brief Illness Perceptions Questionnaire (BIPQ) assessed sickness beliefs. The assessment comprises five items for cognitive representations (consequences, timeline, personal control, treatment control, and identity), two items for emotional representation (concerns and emotional response), and one item for illness comprehension and understanding. The BIPQ concludes with an open-ended question about causal beliefs, where participants rank the three main causes of illness. The Summary of Diabetes Self-Care Activities (SDSCA) is used to assess the frequency of diabetes-related self-management activities in the previous seven days.	Larger networks, more frequent contactors, and higher levels of emotional work received were key predictors of diabetes concern and emotional distress (measured by the BIPQ). The first two factors, network size and contact frequency, appear to contribute primarily to emotional work. According to quantitative data, reported concern, emotional distress, and health outcomes correlated (*p* < 0.05) with specific social network traits, such as emotional and illness work. Emotional work was still a significant predictor of emotional distress and perceived concern about diabetes after multivariate analysis (*p* < 0.05).
Islam et al., 2013, US [29]	26, Bangladeshi, yes	Mixed method,12 Months	21–85, M-11, F-15	NM, 14.4 yrs	Education (Community health worker facilitated session)	The intervention consisted of six monthly 2.5-hour group sessions led by CHWs. The first session provided an overview of diabetes, including information specific to the disease, as well as myths and facts, and an explanation of blood glucose levels. Later sessions covered physical activity, diabetes complications, stress and family support, diet, and health care access. Over a nine-month period, 46 sessions were provided, with an average of five participants per session. During months 3, 6, and 9, study participants received three one-on-one visits from CHWs, each lasting approximately 60 to 90 min. Surveys were gathered during the baseline and follow-up periods. Clinical, behavioural, and participant satisfaction were the outcomes, with qualitative data collected from CHWs.	From baseline to one year later, improvements were observed in health and physical activity self-efficacy, frequency of foot monitoring, knowledge of diabetes, exercise and nutrition for diabetes control, and medication compliance. HbA1c, weight, and body mass index all decreased. Program assessment found the intervention to be highly acceptable.

**Table 3:** Summary of qualitative studies

Author, year and Place of study	Sample, Population, Translation provided or not	Study Design, Analysis & No of Interviews	Age group, Male/Female	Number of years being diagnosed & How long being an immigrant	Self-care/management focus	Self-care/management description	Summary of main findings
Deol et al., 2022, Northern California, US [34]	12, Indian, no	Qualitative study, Content analysis, 2	40–75, M-6, F-6	10.3 ± 6.2, 19.3+/−9.9	Practices	Private, open-ended interviews centred on daily management and practices were conducted with each participant using an interview guide that had been tried and tested in an earlier pilot study. Follow-up interviews were held with each participant to ensure understanding and to complete all study questions. The interviews included questions about the habit of taking Ayurvedic medicines, dietary practices, gender roles, and the need for appropriate diabetes education.	Ayurvedic principles, allopathy, dietary practices, gender roles, inadequate information, and culturally unsuitable diabetes education all had an impact on the balancing act that constituted diabetes self-management. Dietary habits and cultural beliefs affected how AIs treated their diabetes. Allopathic medicine combined with Ayurveda gave participants more control over how they managed their diabetes. Gender, family responsibilities, and a lack of understanding all impact daily diabetes dietary management. Family duties, caregiving tasks, and a lack of support negatively impacted female participants’ ability to manage their diabetes. They had little time to manage their diabetes due to caring for their children, husband, and elderly parents. There was no family conversation regarding the sickness or changes in family behaviours among affected women. In contrast, AI men reported better diabetes control when their spouses made meals.
Wan et al., 2022, Australia [30]	18, Indian, Yes	Mixed method study-Qualitative data, Content analysis, NM	40–63, M-13, F-5	8, NM	Dietary management	In-depth, semi-structured, audio-recorded telephone or videoconference interviews were conducted in participants’ preferred language with an interviewer of a similar cultural background, who had no experience in diabetes management. Interviews focused on dietary practices to manage diabetes, like what foods are good for diabetes management and what foods need to be avoided.	The participants reported not knowing about available organisational support and having trouble implementing diabetes-related food and lifestyle guidelines into their daily routine. They discussed the challenges they faced in obtaining social support from friends and family, and how they relied on medical experts for help. Proficiency in reading dietary information labels and self-blood glucose monitoring techniques was among the facilitators.
Piombo et al., 2020, Italy [31]	55, Bangladesh, Morocco, Algeria, Tunisia, Egypt, Yes	Mixed method study, 2 Follow-ups, 3 &6 months	22–65, M-49, F-6	NM, 11.4 yrs	Culturally tailored dietary intervention	The nutritionist created two distinct semi-structured questionnaires. The baseline questionnaire included a simple food frequency questionnaire comprising 25 items (7 open). The follow-up questionnaire included 16 additional items (7 open-ended) to assess customer satisfaction, diet adherence, and suggested activity levels. A Likert scale, with points ranging from (1) very low to (5) very high. The constant comparative method (CCM) was used to evaluate the incisiveness of the culturally customised intervention.	Numerous interviewees expressed satisfaction with their eating habits, and most stated that they adhered to a normal or similar diet from their country of origin due to “their taste” or “no real free choice,” particularly for those who did not live alone or for limited personal funds. The worst dietary practices were found to be consuming excessive amounts of sugar and generally inadequate amounts of food.
Pardhan et al., 2020, Peterborough and Cambridge, UK [19]	35, Pakistani, Nepalese and Indian, Yes	Qualitative study (Focus group discussions), Thematic analysis, 1	62, M-18,F-17	5->10, NM	Perspectives	In six focus group discussions (FGDs), patients were divided into three groups based on their age (30–60 years, ≥ 60 years), gender (male, female), and literacy level (literate, illiterate). The data were examined iteratively using theme analysis approaches. The areas explored included knowledge and awareness about diabetes and its complications, self-help strategies, and factors that influence self-help and the uptake of available healthcare services.	Various patient populations face distinct obstacles in acquiring more knowledge about diabetes and self-help. For the majority of elderly and illiterate participants, the absence of culturally relevant diabetes education and awareness initiatives in the community seemed to be a significant obstacle, whereas younger participants mentioned time constraints. According to the literate groups, the information provided by healthcare providers was generic and not tailored to their culture and/or dietary needs. Additional obstacles to asking for guidance or assistance included reluctance to reveal their diabetes because it can impact both labour and job (literate populations) and social gatherings (illiterate people), where they may cause anxiety about being singled out. All groups expressed a general lack of enthusiasm for exercise.
Zhang et al., 2018, Auckland City, New Zealand [42]	6, Indians, No 1 pre-diabetes	Qualitative study (Inductive and deductive -Thematic analysis), 1	55–79, M-4, F-2	Pre-diabetes 0.5, DM-11.2, NM	Carbohydrate knowledge and expectations of nutritional support	Skype interviews were done. The questions asked pertained to knowledge of pre- and type 2 diabetes, diabetes support received, advice from clinicians, sources of diabetes and nutrition information, details wanted to know about diabetes nutrition, and healthy and unhealthy foods.	The need for sufficient consultation time with medical practitioners to address participants’ questions and concerns about diabetes management and diet was strongly expressed. Participants knew less about the specific diabetes education services that were offered. Participants desired continuous support and reminders to promote medication adherence and achieve and maintain a balanced diet. The main findings were that people fear complications from diabetes, are willing to participate in managing their condition, need advice to be practical, use simple language, avoid medical jargon, recognise a lack of knowledge and confusion regarding diet and medication, and seek self-directed information despite some degree of mistrust and confusion with websites. When offered trustworthy and culturally relevant advice, participants indicated a readiness to change their diet and sources of carbohydrates. Although some participants self-checked their body weight and blood sugar, there was a widespread consensus that professionals should take these measurements rather than the people themselves. The participants believed that alcohol contained little carbohydrate, while beetroot, cola, rice, soft drinks, and potatoes contained a significant amount.
Abuelmagd et al., 2017, Norway [36]	120, Pakistani women, Yes	Qualitative study, Content analysis, 1	29–80, F-120	Less than/greater than 10 yrs, 28.7	Beliefs and practices	The survey comprises a personal interview, 16 open-ended questions, and 64 closed-ended questions. The primary subjects covered are type 2 diabetes, health status, food and exercise routines, blood glucose monitoring, diabetes medication, and the demand for medical information.	One-third reported poor health, and 71% reported macrovascular comorbidities. The majority reported an inadequate diet, including religious fasting and inactivity. A third were unable to check their blood glucose levels. The diabetes medication regimens varied widely, with one-fourth of patients needing to take insulin in addition to pills. In terms of lifestyle choices, comorbidities, and drug use, Pakistani women living in Norway demonstrated inadequate control over their type 2 diabetes. Low literacy and cultural factors make it challenging for individuals to follow health and lifestyle recommendations.
Patel et al., 2016, London, UK [33]	10, Indian, No	Qualitative study, Thematic analysis, 1	Mean 61, M-8, F-2	NM	Beliefs and behaviours	Semi structured The interviews included questions related to causal beliefs about diabetes, vvvvb adaptation, the use of alternative therapies, diet modification, and sources of information.	The people who were interviewed were aware that they had diabetes, but they found it difficult to change their diet. The utilisation of alternative remedies, moderation in diet, adaptation of exercise regimens, and information sources were among the themes found to be causative beliefs regarding diabetes. Although everyone knew that certain foods should be avoided, some people continued to consume them. People with diabetes indicated a need for forums sensitive to cultural differences.
R Majeed-Ariss et al., 2015, Teesside, England, [35]	15, British Pakistani, Yes	Explorative qualitative study Content Analysis, 1	31–76, F-15	5–29 Yrs, NM	Perception	Face-to-face semi-structured English and Urdu language interviews were conducted with a purposively selected heterogeneous sample of 15 British Pakistani women with type 2 diabetes. The participants were asked mainly about how they felt when diagnosed with diabetes, how they feel now, how their feelings affect diabetes self-care, their role in everyday life, how their role affects their DM Management, etc.	Women reported prioritising their family’s demands over their own as a major impediment to self-management. Ethnically diverse women’s self-identity and diabetes self-management are influenced by their generational status, and culturally competent practice should take this into account. Healthcare providers need to be aware that good self-management occurs in tandem with other facets of life and is influenced by them. Time is another crucial concept. Because their roles constantly changed due to personal or general health changes, patients had to adjust how they identified to continue practising good self-management.
Patel et al., 2015, Manchester, UK [39]	44, Indian, Pakistani, Bangladeshi and Nepali, Yes	Qualitative study Content analysis, 1	32–84, M-23, F-21	0–10 + YRS, NM	Management behaviours whilst holidaying in the East	Semi structured Interviews. The themes explored were differing roles and opportunities for social support networks back home, Beliefs about diet and diabetes management, and the limited role perceived for GPs/practices.	Migrant British South Asians reported a strong inclination to reside in a warmer environment; they felt they had a healthier lifestyle in the East and often altered or abandoned their diabetes treatment. Acquiring diabetes-related information and accessibility to social networks in the East were highly appreciated. In the East, social networks are an important resource for diabetes support and knowledge. Some migrant British South Asians do not follow their prescription regimens when abroad, which may indicate that they do not understand diabetes well.
Patel et al., 2015, North West England, UK [32]	67, Indian, Pakistani, Bangladeshi and Nepali, No	Qualitative study, Content analysis, 1	Mean 61, M-36, F-31	0–10 + yrs, NM	Beliefs	A social network survey, interview, and semi-structured interviews. A social network survey, interview, and semi-structured interviews. The three themes analysed were: fatalism, diabetes management - a family affair, and the use of alternative therapies.	Sociocultural settings appear to influence people’s perceptions of diabetes and their management of the condition. A deeper comprehension of the contextual factors that influence behaviour may make creating culturally relevant therapies that help this population manage themselves and improve their beliefs easier. The qualitative data suggest that fatalistic attitudes and beliefs influence self-management techniques, and people frequently turn to alternative food “therapies” that are often promoted by social networks.
Tang et al., 2013, Vancouver, Canada, [41]	8, English and Punjabi speaking South Asian, Yes	Single cohort study, Content analysis, 3-month training	41–72, M-2, F-6	NM	Education and psychological aspects	A 20-hour peer-leader training programme was conducted over five sessions (4 h per session). The program taught communication, facilitation, and behaviour modification skills through several instructional approaches, including quizzes, group brainstorming, skill-building, group sharing, role-playing, and simulations. To graduate, participants needed to meet competency criteria in four training domains: active listening, empowerment-based facilitation, five-step behavioural goal-setting, and self-efficacy. Participants were allowed three attempts to pass each competency domain.	The results indicate that it is possible to develop and equip peer leaders with the abilities needed to lead a diabetes self-management support group. Since only two of the eight peer leaders in this community had been diagnosed with Type 2 diabetes, it was especially crucial to broaden the concept of “peer leader” to include carers. Another plausible explanation is that the recruitment strategies targeted the general population rather than people with diabetes. It is possible that the diagnosis of diabetes still carries some stigma in the South Asian community, which discourages people with the disease from participating. Following the training program, a qualitative review revealed some conflicting comments. One peer leader suggested that a more extended training session is necessary, and another proposed that diabetes knowledge should be included. Peer leaders questioned further about these remarks and said that a foundational understanding of diabetes education will boost their confidence in a leadership position.
Islam et al., 2013, US [30]	26, Bangladeshi, yes	Mixed method,12 Months	21–85, M-11, F-15	NM, 14.4 yrs	Education (Community health worker facilitated session)	Surveys were gathered during the baseline and follow-up periods. Clinical, behavioural, and participant satisfaction were the outcomes, with qualitative data collected from CHWs.	Qualitative studies revealed that CHWs helped overcome hurdles and promoted program outcomes. It appears that CHWs play a special role in promoting trust in the healthcare system because many participants felt that they could discuss things with their CHWs that they couldn’t discuss with their doctors.
Prinjha et al., 2010, Leicester, UK [40]	67, India, Bangladesh, Sri Lanka, Pakistan, No	Exploratory focus group, Content analysis, 1	18–84, M-26, F 41	NM	Perception	Mobile health SMS text messaging to support medication adherence. Medication adherence is an important component of diabetes self-management. Helping people self-manage their disease can prevent costly and debilitating complications.	According to digital device users, short messages to encourage medication adherence (and other areas of self-management) would be appropriate and relevant. Content that satisfied the participants’ information needs was of special interest to them; examples included information about South Asian meals, frequently used herbs and spices, natural and herbal remedies used in South Asia and the United Kingdom, and religious fasting practices. Short English communications were often considered suitable because family members could interpret them for individuals who couldn’t read or understand them. One way to help patients who have trouble understanding brief English messages is to make them available in several formats and distribute them in person to individuals who do not use digital gadgets. A digital system could also need to incorporate culturally appropriate messages sent to recipients who choose to receive them, thereby satisfying the needs of South Asian people in the UK.
Choudhury et al., 2009, Swansea or Birmingham, UK [37]	14, Bangladeshi, Yes	Qualitative study, Content analysis, 1	26–67, M-4, F-10	6 months-27yrs, NM	Beliefs and practices	The interview was conducted one-on-one in either English or Sylheti. Two separate researchers transcribed and evaluated the interviews. The questions were about the cause of diabetes, preventing diabetes, how they felt when diagnosed with diabetes, diabetes management (including diet, exercise, and medication), information from healthcare professionals, physical activity, information from family or friends, the use of traditional medication, and whether they participated in any diabetes education programme.	Most individuals were unaware of the cause of diabetes. Controlling sugar intake was associated with diabetes management knowledge, and several participants reported using bitter items, such as bitter gourd, to manage their diabetes. Most participants suggested that education sessions should impart knowledge deemed vital by the doctor, and that word-of-mouth advertising would be the most effective way to promote these classes. Participants heavily relied on the doctor’s advice and opinions and were somewhat passive in their self-management. Health practitioners need to be more aware of the activities followed by Bangladeshi individuals, such as consuming bitter gourd, which may intensify the effects of rosiglitazone, and the potential impact these practices may have on diabetes management.
Fleming et al., 2007, Northwest England [38]	9, Indian Gujarati, No	Qualitative, Case study, Content analysis, 2	55–72, M-9	NM	Influence of culture	A case-study methodology was employed, integrating participant observation and interview techniques. Data on Muslim men’s lived experiences with diabetes self-management in Gujarat were gathered. These testimonies, along with additional narrative data from participants, were examined across multiple cycles.	The approach used in various government policies and research, as well as an embodied and dynamic approach to providing culturally appropriate care, are at odds. They argue that the reductionist viewpoint is problematic from both a theoretical and empirical standpoint. They suggest incorporating a dynamic, embodied approach. Such an approach could be used as a framework for generating and implementing culturally relevant research, practice, and policy.
Naeem AG, 2001, Leeds, UK [18]	106, Kashmiri, Yes	Qualitative study, Content analysis, 1	< 40 - >60, M 106	NM	Role of culture and religion	They were interviewed personally in their homes, mosques, community centres, and workplaces. Only Muslim men were interviewed, and they were asked about their living arrangements, marriage to first cousins, their parents’ diabetes status, beliefs about why they have diabetes, health status, economic status, frequency of glucose testing, health status about overweight and blood pressure, and food consumption patterns.	Many men with diabetes were unable to manage their condition, and the general mindset was “leave the rest to Allah.” Even after being told by a doctor that they are obese, they don’t think so. The authors suggested changes in people’s attitudes towards food, activity, blood pressure, and sugar levels. To encourage the consumption of healthy foods, physical activity, and a healthy lifestyle, events such as open days, meetings, and talks should be tailored specifically for men and women.

### Risk of Bias and Quality Assessment

The JBI tool for qualitative studies was used to assess the quality of the 11 articles included in this review. Nearly all studies met at least 70% of the criteria. Three studies (Patel et al., Prinjha et al., and Wan et al.) achieved 90% and met 9 out of 10 criteria, while one study (Tang et al.) achieved 60% and met 6 out of 10 criteria (Figure S1).

For the five cohort studies assessed using the JBI tool’s 11 criteria, three studies (Wan et al., Islam et al., and Patel et al.) met 8 out of 11 items (73%), while two studies (Piombo et al. and Patel et al.) met 6 out of 11 items (Figure S2). The single cross-sectional study (Wan et al.) met 6 out of 8 criteria (75%), as assessed by the JBI tool (Figure S3).

This review included four randomised controlled trials assessed using the RoB V2 scale. The quality check found that all studies had a low risk of bias (Figure S4).

### Findings From Interventional and Observational Studies

The data extracted from the interventional and observational studies are presented in Tables 1 and 2. Two out of three RCTs focused on education interventions [31, 32] and revealed that culturally appropriate education and participant-directed intervention tailoring have a positive effect on glycaemic management and overall scores. An RCT by Vyas et al. found that information was not efficiently conveyed by special interventions such as rotational visits with dieticians, chiropodists, or diabetes nurses [31]. In this study, neither the self-care nor the knowledge awareness scores improved. Another RCT [34], focused on the exercise component of self-care by introducing a one-hour Bollywood dance session; they reported that the intervention group demonstrated a decrease in diabetes parameters.

Similarly, observational data have shown that participation in peer-led or professionally facilitated interventions is associated with better health outcomes, including improved glycemic control [33]. Health worker-facilitated sessions held every six months for 2.5 h nearly improved all diabetes self-care components [35].

Piombo et al. summarised that a culturally tailored dietary intervention significantly improved participants’ nutritional habits [37]. In contrast, Wan et al. analysed the nutrientintakes and the number of servings of each food group consumed by participants over three days. They found that, compared with national dietary recommendations, the median number of servings from the five food groups (Grains, vegetables, fruits, milk, and meats) was lower [36]. Patel et al. examined how sociocultural context affects British South Asians’ perceptions of sickness and diabetes self-care. They found that perceived concern, emotional distress, and health outcomes were associated with specific social network traits (emotional and disease work) [38]. In another study [39], a pile-sorting exercise was conducted using 10 cards, each with a distinct food item written on it, and participants demonstrated a good understanding of which foods are beneficial and which are detrimental to diabetes.

### Findings from Qualitative Studies

Eleven qualitative studies were included in this systematic review (Table 3) [18, 19, 40–48]. The themes focused on exploring various lifestyle practices, perceptions, dietary management, beliefs, and the influence of culture and religion on diabetes self-care. Diabetes self-care behaviours, education, and psychological aspects were also explored in the included studies while holidaying.

Most studies revealed the need for culturally appropriate education and support for all components of self-care [18, 19, 36–40, 42, 43, 45, 47, 48]. One of the significant findings was the key role of diet and culture in the management of diabetes by the American Indians. The knowledge of Ayurvedic principles, ingrained in participants’ daily cultural practices and beliefs, served as the primary foundation for self-treatment [40]. Abuelmagd et al. found in their study that a sizable percentage of participants were unable to self-monitor their blood glucose [42]. Participants were unaware of the causes of diabetes, and their knowledge of diabetes management focused on controlling sugar intake [43]. It was also reported that the impact of culture and religion can impede the right kind of self-care for diabetes [18]. The need for sufficient consultation time with medical practitioners to address participants’ questions and concerns about diabetes management and diet was strongly expressed in another study [48].

One study assessed how mobile health SMS text messaging could improve medication adherence [46]; British South Asians believed that brief messages would be both appropriate and pertinent. They also supported messaging to bolster other facets of self-care [46]. In another study, most respondents reported feeling ignorant and struggling to implement diabetes-related dietary and lifestyle guidelines into their daily routines [36].

Women reported prioritising family demands over their own care [41]. Care-giving tasks and a lack of support negatively impacted their self-care behaviours, whereas men reported better diabetes control when their spouses made meals [40].

Pardhan et al. [19] reported that, for most elderly and illiterate participants, the absence of culturally relevant diabetes education and awareness initiatives in the community seemed to be a significant obstacle, whereas younger participants mentioned time constraints. According to the literate groups, the information provided by healthcare providers was generic and not tailored to their culture and/or dietary needs. Additionally, all groups expressed a general lack of enthusiasm for physical activity.

### Mixed-Methods Data Integration

Table S2 shows the shared concepts underlying both the qualitative and quantitative data. PIP results in three main pillars: providing sufficient education about diabetes self-care and management, involving patients in designing their self-care plans, and integrating interactive physical activity into their daily routines.

In a single-method interventional study, Natesan et al. demonstrated that culturally appropriate dance is an effective intervention for HbA1c management [40]. Similarly, qualitative studies reported a lack of culturally suitable exercise options as a barrier to following recommended physical activity [18], a lack of enthusiasm for exercise [19], and difficulties in adapting to exercise routines [45].

In mixed-methods studies, the qualitative and quantitative sections investigate different aspects. For instance, Wan et al.’s qualitative study explored the participants’ eating habits. In contrast, their quantitative study measured the number of servings and nutritional intake in each food group over the past three days [42]. Patel et al. examined participants’ beliefs and behaviours in the qualitative section. Conversely, in the quantitative part, they used a pile sorting exercise to assess participants’ ability to distinguish between beneficial foods and those that are harmful for people with diabetes [45]. None of the mixed-method studies included in this review provided insight into the participants’ experiences of the research interventions implemented and examined in their study.

## Discussion

This systematic review evaluated 20 studies spanning a broad range of evidence to explore how South Asians living in Western countries manage their diabetes. This review highlighted that providing adequate education and training on various aspects of diabetes self-care, such as dietary management and foot care, has a positive impact on patient outcomes [32, 33]. Interventions aimed at promoting physical activity were also beneficial [34]. However, findings from qualitative studies revealed several challenges. Many South Asian immigrants struggled to adopt recommended lifestyle modifications [36], often because they found it challenging to integrate these changes into their daily lives. Language barriers further hindered their understanding of healthcare information [19, 49]. In addition, some individuals lacked the skills and confidence to independently monitor their blood glucose levels, indicating a broader issue of self-care or management [42].

The self-care practices of individuals from Indian, Pakistani, Bangladeshi, Nepali, and Sri Lankan backgrounds exhibit notable distinctions, primarily shaped by cultural, religious, and socioeconomic factors. For example, Indians often incorporate traditional Ayurvedic practices and modern medical treatments in diabetes management [40, 47]. This approach reflects the widespread cultural reliance on traditional remedies and alternative medicine. Similarly, Lawton et al. [50] in a study to explore the perceptions and experience of taking oral hypoglycaemic agents (OHAs) found that popular beliefs about medications on the Indian subcontinent may influence how Indian and British Pakistani patients view their OHAs. Cultural considerations must be taken into account to ensure these patients receive appropriate advice and prevent unnecessary prescription adjustments. Conversely, Pakistanis frequently emphasise religious practices, such as fasting during Ramadan, which can disrupt consistent self-care routines such as medication adherence and dietary management [18]. Studies conclude that culturally competent care is essential for these populations, particularly in addressing the interplay between traditional beliefs and biomedical practices [32, 48].

Fagerli et al. [51] reported that following the advice of health care providers caused great concern among the participants. However, there were many explanations regarding limitations. These addressed many life-situational concerns, but more significantly, they involved communication issues stemming from gaps between culturally defined lay knowledge and universalising medical knowledge. The individual with diabetes was left to translate across various levels of knowledge because the guidance was typically viewed as insufficient, given the participant’s food and cultural background.

Bangladeshis often encounter language barriers and challenges in accessing healthcare, which impede effective self-management [19, 52]. Many Bangladeshi participants in qualitative studies reported relying heavily on doctors’ advice and exhibited limited active involvement in their care, often due to low health literacy [43]. Additionally, their dietary practices, dominated by staple foods such as rice, pose challenges to glycaemic control [37]. In contrast, Sri Lankans exhibit a greater focus on structured self-care, likely due to government-led health education initiatives in their country of origin. However, as immigrants, they still struggle with adapting to Western dietary norms, which can hinder diabetes management [31].

Social and familial support also varies significantly across these groups. Pakistanis and Bangladeshis tend to rely heavily on familial networks for care, which can sometimes conflict with professional medical advice [19, 53]. Indians and Sri Lankans, on the other hand, are more likely to engage in peer-supported or community-based interventions, such as culturally tailored exercise programs [34]. These differences underscore the need for healthcare interventions to be tailored not only to cultural norms but also to the specific socio-economic realities of each group, ensuring improved diabetes outcomes.

### Integration of Quantitative and Qualitative Findings

Adequate education about diabetes self-care and management is one of the three pillars of achieving optimal diabetes self-care. One RCT found that a one-on-one, structured diabetes health education intervention by a link worker significantly improved participant knowledge [32]. Additionally, a significant portion of participants in the qualitative studies were unaware of the causes of diabetes, and most believed that taking education classes would be beneficial for following the recommended self-care behaviours [36, 42–44, 47].

The second pillar was the importance of incorporating patients into self-care plans. According to a qualitative study [36], participants reported that a lack of social and familial support limited their ability to incorporate dietary and lifestyle recommendations into their daily routines. Since each patient has unique circumstances, an RCT that tailored an individualised care plan led to significant improvements in HBA1C levels [33].

The third pillar was using interactive methods to incorporate physical activity into daily routines. According to a qualitative study [19], most participants reported being physically inactive. One RCT demonstrated that twice-weekly, one-hour Bollywood dance sessions resulted in a decrease in HbA1c levels from baseline [34]. Exercise or physical activity is one of the most crucial aspects of diabetes self-care; when participants incorporate it in various interactive and engaging ways, it can improve their blood glucose levels and adherence to self-care behaviours.

### Optimal Diabetes Self-Care

**Fig. 2:**
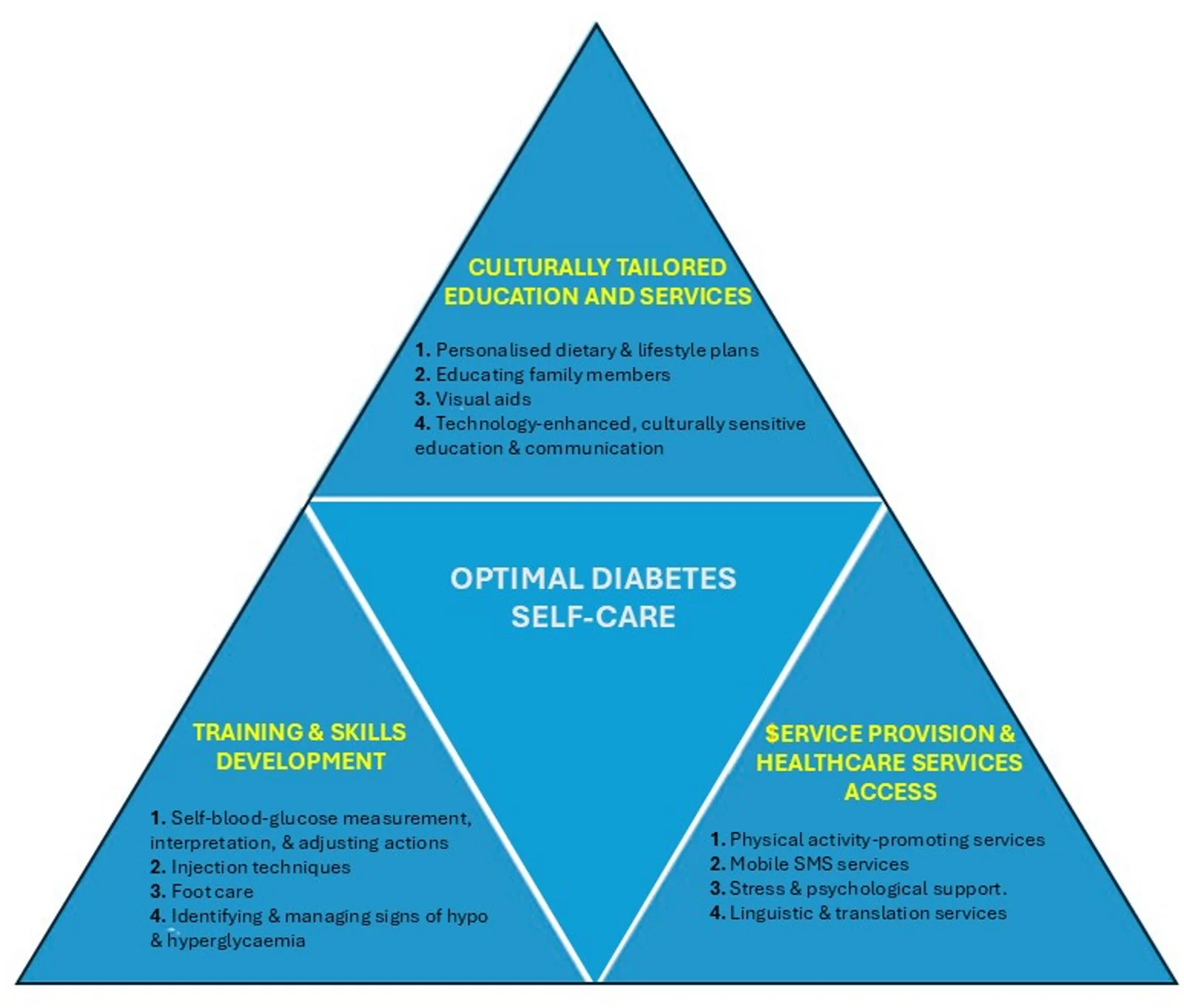
Diagrammatic representation of strategies for achieving optimal diabetes self-care

After analysis of the included studies, we developed a framework (Fig. 2) that integrates three essential domains or strategies: culturally tailored education, training and skill development, and service provision or access to healthcare.

### Culturally Tailored Education and Services

The initial key to achieving optimal diabetes self-care is culturally tailored education. Since South Asians have distinct cultures, beliefs, and lifestyles, it is essential to educate them in a culturally sensitive and appropriate manner to achieve maximum participation. The goal is to develop personalised diabetes care plans that align with the patient’s lifestyle, cultural practices, and preferences. For example, personalised dietary plans accommodate and respect cultural food preferences and traditional ingredients while ensuring nutritional balance. This includes considerations for religious practices, such as fasting during Ramadan and dietary restrictions, including vegetarian or vegan diets, in some cultures.

In many cultures, family plays a significant role in health decision-making [48, 53]; therefore, educating family members is equally important for providing better support and ensuring adherence to self-care practices. In some communities, diabetes may be stigmatised, making patients reluctant to discuss their condition. In such situations, one-on-one counselling can help reduce feelings of isolation and encourage open discussions, leading to improved self-care and management.

Utilising culturally sensitive translators, health educators, or digital platforms tailored to patients’ linguistic and cultural needs can significantly improve information processing and engagement with diabetes management.

### Training and Skills Development

Many participants lacked the skills to administer insulin, care for their feet, and monitor glucose levels. Abuelmagd et al. found that a third of the study population could not check their blood glucose independently [42]. This highlights the importance of training on using glucose meters, understanding trends and target ranges, interpreting the results, and adjusting actions (e.g., insulin units) based on those results. In addition to glucose monitoring, patients need to develop the skills to identify signs of hypoglycaemia or hyperglycaemia and to take appropriate action in these situations. They also need training to inspect their feet for signs of infection regularly and to understand proper foot care.

### Service Provision and Accessible Healthcare Services

Diabetes care is multifaceted and requires access to many medical specialities such as endocrinology, cardiology, nephrology and surgical services. Likewise, several allied health services, such as optometry, podiatry, dietetics, and physiotherapy, also play a crucial role in the optimal management of diabetes. Our review found that patients were supportive of an SMS service reminding them to take their medicines [46]. Another study revealed that, to promote medication compliance and achieve and maintain a balanced diet, participants desired continuous support and reminders [48].

Given how widespread diabetes-related stress is, patients must receive psychological support in any form to adhere to the instructions. As immigrants, amid all other difficulties, migrants strive for stability and dignity while navigating a challenging terrain of barriers in their pursuit of a better life. Therefore, it is essential to ensure that at least their health-related distresses are appropriately addressed. Also, studies showed that diabetes self-care is not the same for men and women in this population, prioritising family’s needs and care above their own, and a lack of support from family negatively influences the self-care patterns of women [40, 41], so it may be beneficial if gender specific counselling, advice and recommendations are provided for this population.

Exercise is a crucial aspect of diabetes self-care, yet research indicates that many participants lack the incentive or motivation to maintain regular physical activity [54]. To address this, some services focus on making exercise more enjoyable and culturally relevant. Developing physical activity-promoting interventions incorporating culturally familiar activities, such as dance lessons, group jogging or walking, or traditional exercises (e.g., Tai Chi), can enhance engagement. Fostering a sense of community among individuals facing similar challenges can provide motivation, emotional support, and accountability, ultimately encouraging long-term adherence to an active lifestyle.

Along with providing adequate services, ensuring they are properly advertised is also mandatory to make people aware of them. One study in the Indian population reported that participants knew less about the specific diabetes education services offered [48].

Culturally-trained staff providing diabetes care can educate patients on accessing healthcare services and support programs, empowering them to seek timely medical care and preventive services.

### Practice Implications

This review’s integrative framework provides a practical guide for healthcare professionals and community health workers aiming to improve diabetes self-care among South Asian immigrants in Western contexts. The three pillars can be operationalised through structured, replicable interventions. For example, the education pillar supports the design of culturally adapted DSMES programs using visual tools, multilingual materials, and community educator models that reflect patients’ dietary customs and belief systems. The patient-centred planning pillar encourages collaborative goal-setting that respects religious practices, caregiving burdens, and gendered dynamics, particularly for women who deprioritise their own care. The third pillar, promoting accessible physical activity, can be implemented through community-based group exercise tailored to cultural norms, such as Bollywood dance sessions or faith-based wellness groups.

The framework also enables health systems to audit and redesign care models to address identified barriers, language proficiency, low health literacy, access issues, and sociocultural mismatches, using the outer ring of the diagram as a diagnostic tool. Teams can use this visual to map local service gaps, inform staff training needs, or co-develop culturally safe interventions in collaboration with South Asian communities. Ultimately, this model summarises evidence and serves as a translational tool that can be embedded into primary care, health promotion programs, or immigrant health services to drive more equitable and effective diabetes care.

Given that most interventional, observational, and qualitative studies demonstrate the benefits of culturally tailored physical activity for patient outcomes, expanding access to such programs at scale could promote healthier lifestyles across diverse ethnic groups. One such example is the SilverSneakers program in the US, funded by Medicare, which provides free or low-cost access to fitness centres and community-based exercise classes nationwide [55]. These initiatives are equally valuable for individuals who are not overweight or obese, as the global burden of type 2 diabetes attributable to non-high BMI accounts for a significant portion of cases, particularly in Asia and other low- and middle-income regions [56]. Therefore, diabetes management and public health measures should not assume that patients are overweight or obese [56]. Instead of relying solely on insulin-sensitising therapies, interventions that increase insulin secretion or utilise exogenous insulin, such as regular physical activity, may be more effective [57]. In people with low BMI, dietary strategies should prioritise maintaining or increasing muscle mass through adequate protein intake. At the same time, physical activity should emphasise resistance or strength-based training to support muscle preservation and metabolic control [58, 59].

Another important aspect is the presence of disparities in access to diabetes care across developed Western countries. Some contributing factors include socioeconomic inequality [60], where populations face limited access to healthcare services [61]. Insurance coverage and reimbursement policies related to newer therapies, such as GLP-1 Receptor agonists [62], also contribute to these disparities. Even within universal healthcare systems such as the NHS, participation in diabetes self-care programmes remains low due to limited awareness and logistical challenges [63].

### Strengths and Limitations

This systematic review comprehensively integrates qualitative and quantitative data examining diabetes self-care practices among South Asian immigrants in Western countries. A key methodological strength of this review is the use of the Pillar Integration Process (PIP), which enabled the structured synthesis of findings across study designs. Through this mixed-methods approach, the review identified three practical, evidence-based strategies to enhance diabetes self-care in this population: culturally tailored education, patient-centred care planning, and interactive physical activity promotion.

However, several limitations should be recognised. Firstly, in managing T2DM, understanding self-care among individuals on insulin is essential; however, this area receives limited attention, so we cannot explore it further. Second, there is a risk of selection bias because only studies published in English were included. Third, the included studies were conducted in different countries with varying healthcare systems, cultural norms, and policy environments, which may limit the generalisability of the findings. Finally, not all Western countries are represented in the evidence base, as some regions yielded no eligible studies during the literature search.

## Conclusion

This review assesses self-care behaviours and practices of South Asians in Western countries and summarises key findings from various studies. Using the Pillar Integration Process, three distinct pillars in diabetes self-care were identified: culturally appropriate diabetes education, patient-centred care planning, and engaging, accessible physical activity interventions. Education-focused interventions, especially when culturally adapted, have a positive influence on diabetes knowledge and self-care. Cultural beliefs, language barriers, and family roles have a significant influence on diabetes self-care. Women, compared with men, faced difficulties prioritising their health due to caregiving responsibilities. Incorporating these factors into diabetes management could boost patient engagement, improve diabetes outcomes, and support better long-term self-management within this population.

## Supplementary Information

Below is the link to the electronic supplementary material.

Supplementary Material 1

## Data Availability

No datasets were generated or analysed during the current study.
