# Supplementary material for: Diabetes Self-Care among South Asians in Western Countries: A Systematic Review
Source: J Immigr Minor Health. 2025 Nov 20;28(5):1246–67. doi: 10.1007/s10903-025-01825-4 (PMC13612628; doi:10.1007/s10903-025-01825-4)
Supplement: Supplementary file 1 [file 10903_2025_1825_MOESM1_ESM.docx]

|  | Yes | No | Unclear |
| --- | --- | --- | --- |
| Deol et al | 70% | 10% | 20% |
| Abuelmagd et al | 80% | 20% | 0% |
| Choudhury et al | 70% | 20% | 10% |
| E.Fleming et al | 80% | 0% | 20% |
| A G Naeem | 70% | 20% | 10% |
| Patel et al | 90% | 10% | 0% |
| Patel et al | 80% | 20% | 0% |
| Patel et al | 70% | 20% | 10% |
| Prinjha et al | 90% | 0% | 10% |
| Tang et al | 60% | 20% | 20% |
| Wan et al | 90% | 0% | 10% |
| R Majeed-Ariss et al | 80% | 20% | 0% |
| Zhang et al | 80% | 20% | 0% |
| Pardhan et al | 80% | 20% | 0% |
| Islam et al | 80% | 20% | 0% |
| Piombo et al | 80% | 20% | 0% |

*Figure S1: Quality assessment using the JBI Critical Appraisal tool for qualitative studies*.

|  | Yes | No | Unclear | Not Applicable |
| --- | --- | --- | --- | --- |
| Wan et al | 72.7273% | 0% | 9.09091% | 18.1818% |
| Islam et al | 72.7273% | 0% | 0% | 27.2727% |
| Piombo et al | 54.5455% | 0% | 9.09091% | 36.3636% |
| Patel et al | 54.5455% | 0% | 0% | 45.4545% |
| Patel et al | 73% | 0% | 0% | 27% |

*Figure S2: Quality assessment using the JBI Critical Appraisal tool for Cohort studies*

|  | Yes | No | Unclear |
| --- | --- | --- | --- |
| Wan et al | 75% | 0% | 25% |

*Figure S3: Quality assessment using the JBI Critical Appraisal tool for cross-sectional study*

| **Study** | **Randomisation process** | **Deviations from intended interventions** | **Missing outcome data** | **Measurement**  **of the outcome** | **Selection of the reported result** | **Overall, Bias** |
| --- | --- | --- | --- | --- | --- | --- |
| **A Vyas et al** | + | + | + | + | + | + |
| **Hawthorne** | - | + | + | + | + | + |
| **Tang et al** | + | + | + | + | + | + |
| **Natesan A et al.** | + | + | + | + | + | + |

*Figure S4: Quality assessment of RCTs using Cochrane ROB V2 assessment scale.*

| + | Low risk |
| --- | --- |
| - | Some concerns |

**Table S1:** Inclusion and exclusion criteria using the SPIDER framework.

| **SPIDER** | **Inclusion** | **Exclusion** |
| --- | --- | --- |
| Sample | South Asian origin immigrants of all age groups diagnosed with type 2 DM | Studies focused on other conditions and type 1 DM |
| P of I: Phenomenon  of interest | Studies examined self-care practices such as a diabetic diet, self-glucose monitoring, medication adherence, exercise, and others. RCTs examined the effectiveness of any self-care-improving interventions, such as education or participant-directed intervention tailoring. Qualitative studies exploring perceptions and beliefs about diabetes self-care were included. In addition, qualitative studies that analysed the role of culture and religion in self-care were also considered. | Studies dealing with other aspects of diabetes management |
| D: Design | Original research articles with experimental and observational or qualitative or mixed-methods designs, such as cross-sectional, cohort, case-control, and randomised control studies | Systematic reviews, meta-analyses, conference presentations, and letters of correspondence |
| E: Evaluation | Effects of self-management interventions, Content Analysis of qualitative studies  Outcomes of interest  • Educational and psychological aspects  • Beliefs and Daily Practices  • Knowledge and awareness  • Exercise or physical activity  • Perceptions about diabetes self-care  • Role of culture and religion  • Dietary management  • Microaggression and diabetes distress  • Acculturation and Glycemic Control  • Sociodemographic, lifestyle, & cultural factors  • Role of social networks and beliefs | No self-care-related or other disorder-related outcomes |
| R: Research | Qualitative, quantitative and mixed-method studies  Articles published in the English language between January 1, 2000 and December 31, 2024 | Non-English language studies published before 2000  Studies full texts are not available  Population not specified |

Table S2: Pillar integration

| **Quantitative Data** | **Pillar** | **Qualitative data** |  |
| --- | --- | --- | --- |
| Nearly everyone improved their knowledge and glycaemic control after one-to-one structured diabetes health education delivered by a link worker with pictorial flash cards and visual aids. | Providing adequate education about all aspects of diabetes and its management. | The majority of participants did not know what caused diabetes. Knowledge of diabetes management was linked to controlling sugar intake, and several participants reported eating bitter foods such as bitter gourd to control their diabetes. There was little access to information, as many participants did not speak English or have a Bengali-speaking doctor. Most participants felt that education classes should teach them what the doctor thought was important, and that these classes would best be advertised by word of mouth. |  |
|  |  |  |  |
|  |  |  |  |
|  |  |  |  |
| Participate-directed intervention tailoring improved the HBA1C level significantly. | Involving patients in designing a self-care or management plan | Participants reported difficulties incorporating diabetes-related dietary and lifestyle recommendations into their routine and a lack of knowledge about available organisational support. They mentioned challenges in receiving social support from families and friends, and relied on support from health professionals. Facilitators included proficiency in nutrition information label reading and self-blood glucose monitoring skills. |  |
|  |  |  |  |
|  |  |  |  |
|  |  |  |  |
| One-hour physical activity (e.g., Bollywood dance classes) offered twice weekly demonstrated a decrease in Ab A1c from baseline. | Incorporating interactive physical activity or intermittent fasting into a daily routine | A majority reported physical inactivity and an unhealthy diet, including religious fasting. |  |

Table Search strategy and results

| **Database** | **Date started** | **Keywords** | **Filters** | **Overall number of articles before screening** |
| --- | --- | --- | --- | --- |
| Medline | 31 March 2024 | Type 2 diabetes or type 2 diabetes mellitus or t2dm or t2dm or NIDDM or non-insulin dependent diabetes mellitus’, ‘self-management or self-care or self-regulation or self-monitoring’, ‘South Asian or Asian or Bangladeshi or Bengali or Gujarati or Indian or Srilankan or Punjabi’, ‘British south Asian’ and ‘South Asian immigrants | Peer reviewed  Date: 1^st^ Jan 2000 to 1^st^ Jan 2024  English Language  Human  All Adult 19+years | 221 |
| PsycINFO | April 1 2024 | ‘Type 2 and diabetes’  ‘Diabetes and self management’  ‘Diabetes and self-care’  ‘Immigrants’  ‘South and Asian’ | 1^st^ Jan 2000 to 1^st^ Jan 2024 | 51 |
| CINAHL | 31 March 2024 | ‘type 2 diabetes or type 2 diabetes mellitus or t2dm or t2d or niddm or non-insulin dependent diabetes mellitus’, ‘self care or self-care or self-management or self management’ and ‘south asian or asian or bangladeshi or bengali or gujarati or indian or sri lankan or pakistani or punjabi | Peer reviewed  Date: 1^st^ Jan 2000 to 1^st^ Jan 2024  English Language  Human  All Adult 19+years | 96 |
| Central | 2 April 2024 | ‘Type 2 diabetes’  ‘Self-care’  ‘Self- management programme’  ‘Immigrants’  ‘South Asian’ | 1^st^ Jan 2000 to 1^st^ Jan 2024 | 281 |
| CTgov | 2April 2024 | ‘Type 2 diabetes’  ‘Self-care’  ‘Self- management programme’  ‘Immigrants’  ‘South Asian’ | 1^st^ Jan 2000 to 1^st^ Jan 2024 | 705 |
| ICTRP | 2 April 2024 | ‘Type 2 diabetes’  ‘Self-care’  ‘Self- management programme’  ‘Immigrants’  ‘South Asian’ | 1^st^ Jan 2000 to 1^st^ Jan 2024 | 501 |
